# Intonation and timing in singing early music is unrelated to respiration synchronization

**DOI:** 10.1038/s41598-026-39565-6

**Published:** 2026-02-27

**Authors:** Anton Schreiber, Klaus Frieler, Elke B. Lange

**Affiliations:** Max-Planck-Institute for Empirical Aesthetics, Frankfurt, Germany

**Keywords:** Music performance, Singing quality, Respiration synchronization, Empirical aesthetics, Hyperscanning, Neuroscience, Physiology

## Abstract

Musical ensemble performance offers a unique context for investigating complex interpersonal coordination. Hyperscanning research revealed the synchronization of physiological processes during coordinated musical interaction. In a previous study, we showed that physical touch enhanced respiratory coupling between professional singers performing together. We expand on this research by investigating whether this respiration coupling affects performance quality in terms of timing and intonation. Eight singers (two for each voice part) performed Renaissance works multiple times, both with and without physical touch. We annotated the recorded vocal tracks with precise onset times and pitch information, resulting in 64,214 tone events. We analyzed timing as mean onset deviation and intonation as mean absolute pitch deviation, both within voice groups. In contrast to the findings on respiration coupling, physical contact had no effect on timing and intonation, indicating that there is no relation between respiration coupling and singing quality. When tested directly with linear mixed effect models, respiration coupling did not predict intonation or timing in five of six models. One model showed a negative effect: higher respiration coupling decreased intonation accuracy. We conclude, in a professionally trained vocal ensemble, increased respiration coupling does not enhance singing quality.

**Supplementary Information:**

The online version contains supplementary material available at 10.1038/s41598-026-39565-6.

## Introduction

 Ensemble music performance is one example of highly refined social interactions between humans^1–3^. It requires complex and joint action coordination by non-verbal communication^1,3,4^ and enables studying the involved neurological and cognitive processes in ecologically valid settings^1,5^. Hyperscanning methods, i.e., synchronous data acquisition of time-based data of two or more interacting subjects, revealed coupling of neural and physiological processes between musicians performing together^5^. However, the artistic consequences of such physiological coupling have not been investigated so far. In this study, we extend the current research by evaluating the relation between the synchronization of respiration signals and the quality of the singing performance.

A growing body of research applies hyperscanning methods on music performance (for a review, see^6^. Studies have investigated a variety of musical constellations from guitar ensembles^7–9^, piano duos^10–13^, vocal ensembles and choirs^14–22^, and saxophone ensembles^23–25^. While some studies focus on neural correlates and inter-brain synchronization (IBS) during music performance^6,20,23–27^, others investigate physiological synchronization of respiration and heart rate variability (HRV)^15–19,21,22^.

In the first hyperscanning study on singing performance, Müller and Lindenberger showed synchronized cardiac and respiratory coupling between singers of a choir while singing compared to a silent resting condition^19^. Synchronization was higher when singers sang in unison (i.e., the same) compared to a canon (i.e., singing the same melody shifted in time). Synchronization of these physiological processes in ensemble singing has been replicated in varying conditions^15–18,22^. Also, HRV synchronized when simply vocalizing together and was strongly influenced by breathing^21^. One study systematically varied coordinated breathing (i.e., inhalation) of singers in three experimental conditions: humming, singing a mantra, and singing a hymn^22^. Analogous to^19^, HRV increased in all three conditions compared to a silent baseline. Compared to humming with unguided breathing, the synchronized breathing during the hymn and the mantra performance increased synchronization of HRV between singers. This indicated that HRV synchronization between singers is based on the coupling of respiration and HRV via the respiratory sinus arrhythmia (see also^21)^. That is, interpersonal physiological synchronization increases by inhaling at the same time. Importantly, when singing, the musical structure imposes preferred time points of inhalation in pauses or in between musical phrases. The question arises whether physiological coupling during singing occurs when synchronized inhalation is avoided. Indeed, professional singing ensembles can coordinate their breathing and stagger inhalation between or within voices to maintain a continuous flow of music. One study showed that even under these conditions, both respiration signal and HRV were synchronized between the members of the singing ensemble, but the coupling index was lower than in other studies^16^. Singers performed polyphonic music, which is composed of differently timed voices compared to monophonic (single voice) or a strict homophonic texture. Respiration coupling expanded to passages of the music where one or more voices rested while others continued singing^16^, revealing a higher level of organization between performers through music^28^.

The underlying theoretical framework for hyperscanning studies in the musical context comes from the field of social action coordination^28–30^. For example, the joint forward model for interpersonal action coordination^28^ assumes three levels of representations of actions (self, others) and coordination (own motor control, joint goals, joint actions): Each individual represents their own motor control on one level (e.g., singing from a score). If a group of individuals shares the same goal (e.g., performing music together), the others’ forward models are represented as joint goal on a second level, accompanied by a joint forward model for the joint action on the third level. Empirical support comes from network analyses of physiological signals and musical performance data (e.g., HRV, respiration and within- and cross-frequency synchronization), demonstrating complex interactions of signals within and between musicians performing together^14,26–28^. For example, analyzing connectivity strength in networks of within-frequency and cross-frequency couplings revealed that the respiration signal of choir members singing the third entry was related to other members’ voice signal of the second entry^17^. That is, physiological signals synchronized not only when humans shared similar processes (singing and listening to the same music, inhaling at the same time), but also by coordination of different actions (singing different parts as in^17,19^, singing versus listening as in^16^. This strongly indicates, that a group of individuals can function as a *superorganism*^14,28^, based on principles of self-organization and circular causality. When singing in a choir, the individual singer contributes to the superorganism in a local-to-global direction, while the choir as a whole constricts the individual performance in a global-to-local direction^14^.

In our study, we are interested in the consequences of synchronization of physiological processes for the artistic quality of singing performance. Some previous research related physiological or neural synchronization and audio recordings of musical performances, but with different foci. For example, the recorded sounds of guitars^26,27^ or voices of singers^17,18^ have been analyzed analogously to physiological processes, differentiating frequencies in the signal by wavelet transformation and creating a normalized signal. By such a transformation, signals from different domains (behavioral, neural, physiological) were incorporated into the same analyses (e.g.,^14^, revealing coordinated dynamics between signals. In another study, using different methods, motor synchronization in piano duets was manipulated and analyzed through onset differences of key strokes^30^. Behavioral synchronization was associated intra-individually with suppressed alpha oscillations in the brain. In other studies, with piano duets^10–13^, interbrain-synchronizations were investigated. When pianists planned similar tempi during a pause, inter-brain synchronization increased^10^, indicating that similarities of cognitive processes affected neural synchronization in the absence of sensory input or movement execution. The neural synchronization did not increase synchronization of the first keystroke after the pause, additionally questioning a strict relation between synchronization levels. A further study^11^ showed that there was higher brain synchronization between two pianists during phases, when one pianist disturbed the performance by changing the tempo unforeseeably. That is, the brains synchronized in phases, when pianists tried to align themselves but were not yet aligned temporally. In another study^13^, one pianist was assigned the role of a leader and changed the tempo during playing together. The follower-pianist showed overlapping spectral peaks of brain amplitudes with the leader, related to the tempo of the leader’s motor performance. In another study^31^, musical laypersons, who were not familiar with each other, had to play drums together. Their intention to play togetherࣧ rather than actually drumming in synchronyࣧ increased physiological synchronization. Together, the studies showed that physiological or neural synchronization can occur without synchronized motor performance or shared sensory input. However, common sensory input and joint motor performance can align neural and physiological processes between performers^12,13,19,22^.

Importantly, to our knowledge, no study has yet looked directly into the quality of musical singing performances in relation to physiological coupling, which is the focus of our study. However, the above mentioned studies indicated that the relation between physiology and performance depends on the task context. When an ensemble sings in polyphony, the musicians need to integrate information from self and others^28,29^. The attentional focus has to change dynamically between perceiving and evaluating the performance of the others, or the own, in order to adapt the own performance to achieve personal and group goals. In this context, it seems relevant that studies on joint drumming or decision making showed a positive effect of physiological synchrony on self-reported group cohesion^32^. Reported cohesion might also relate to the ensemble’s singing performance and its quality, which can be quantified by measuring the singers’ ability to adjust intonation (matching pitch between singers) and timing (synchronous onsets of tones) within the ongoing process of joint music making. Moreover, respiration as a physiological signal is a relevant measure in the context of singing, since singers must finely regulate their breathing. Singers adjust both inhalation and exhalation to produce pitch, onset, duration, and loudness of each musical note^33^, and the phrasing of the overall musical structure. Producing sound is a highly complex process^33^. Vocal fold vibration requires subglottal pressure, and the minimum subglottal pressure required to initiate vibration, known as phonation threshold pressure, is determined by laryngeal biomechanics such as vocal fold adduction, mass, and fundamental frequency. The actual subglottal pressure during singing is controlled by coordinated action of the diaphragm and abdominal muscles, and this fine motor control affects the quality of the output. For example, professional singers show greater abdominal contribution than untrained singers while singing^34^. In sum, singing depends strongly on complex movement control of the respiratory apparatus. It is therefore highly likely, that synchronization of the respiratory signal in ensemble singing has a positive effect on the quality of coordinated singing in terms of intonation and timing accuracy.

The current study builds on a previous one^16^ by investigating the artistic consequences of interpersonal synchronization of physiological processes. Inspired by a question from historical performance practice, the experimental design in^16^ included recordings from three different spatial arrangements of the ensemble on stage: i) modern performance practice (“modern”); (ii) standing close with physical contact via putting the hands onto the neighboring singers (“touch”); (iii) standing close without physical contact (“no touch”). It was shown that synchronization of the respiration signal increased by physical touch between singers, by comparing the mean coupling index (each singer’s coupling with all other singers) between the arrangements (ii) and (iii).

The unique conditions of physical contact in this experimental setting support a further argumentation in favor of increased singing accuracy. Vibrations of the body caused by singing^35^ might serve as a form of somatosensory information transfer between singers. In fact, vibrotactile feedback has been demonstrated to support perception of pitch and timbre^36^. Therefore, the physical contact might facilitate the transmission of vibrational cues between ensemble members, which increases the accuracy of singing performance. Further supporting evidence comes from studies on increased synchronization of motor behavior via physical contact^37–39^.

In the current study, we evaluated singing quality in terms of intonation and timing from the audio recordings and related those measures to the synchronization of the respiration signal, as already published in^16^. Musical stimuli were four-part vocal compositions from the Renaissance, where each voice is sung by two singers in unison. Perfectly strict timing is usually no stylistic goal in music performance, as deviations in micro timing are characteristic for artistic interpretations^40,41^. For timing, we therefore decided to measure deviations of tone onsets between the two singers of the same voice. Likewise, for intonation, we measured deviations of pitch between the two singers of the same voice. An alternative measure for pitch accuracy is to compare the performed note with the score (e.g.,^42^) , but this has several shortcomings in our context. First, this depends on the tuning system employed by the singers (e.g., equal temperament, just intonation) as well as it is subject to an overall pitch drift which is not uncommon^43–45^. Hence, we capitalized on the fact that two singers were always singing in unison in each voice, to directly measure musical synchronization in the pitch and time domain. Respiration coupling was analyzed by three measures commonly reported in the literature^8,15,16,19^: the Absolute Coupling Index (ACI), the Phase Synchronization Index (PSI), and the Integrative Coupling Index (ICI) (see^16,19^ for more details on the differences between measures).

To test for an effect of respiration synchronization on singing quality, we applied two approaches: (1) We compared singing quality between the two conditions: with and without physical contact, which affected respiration synchronization^16^ and may impact singing quality via vibrotactile feedback. Analyses based on paired *t*-tests (aligned to^16^, or linear mixed-effect models including the two conditions as contrast. For the *t*-tests, averaged data included 32 observations. For the models, we used the smallest units of performance on the piece level with 224 observations. Both approaches are indirect tests of a relation between respiration synchronization and singing performance, as we compare singing quality in two conditions that are known to differ in respiration synchronization^16^. (2) The second approach tested directly, whether respiration synchronization predicts singing quality. To gain statistical power, we took data from all three spatial arrangements of the ensemble (i−iii) into account. We calculated linear mixed effect models predicting the dependent measures by the coupling strength across recordings. To align measures, data were averaged on the level of the units for the respiration analysis, resulting in 92 observations.

## Results

**Fig. 1:**
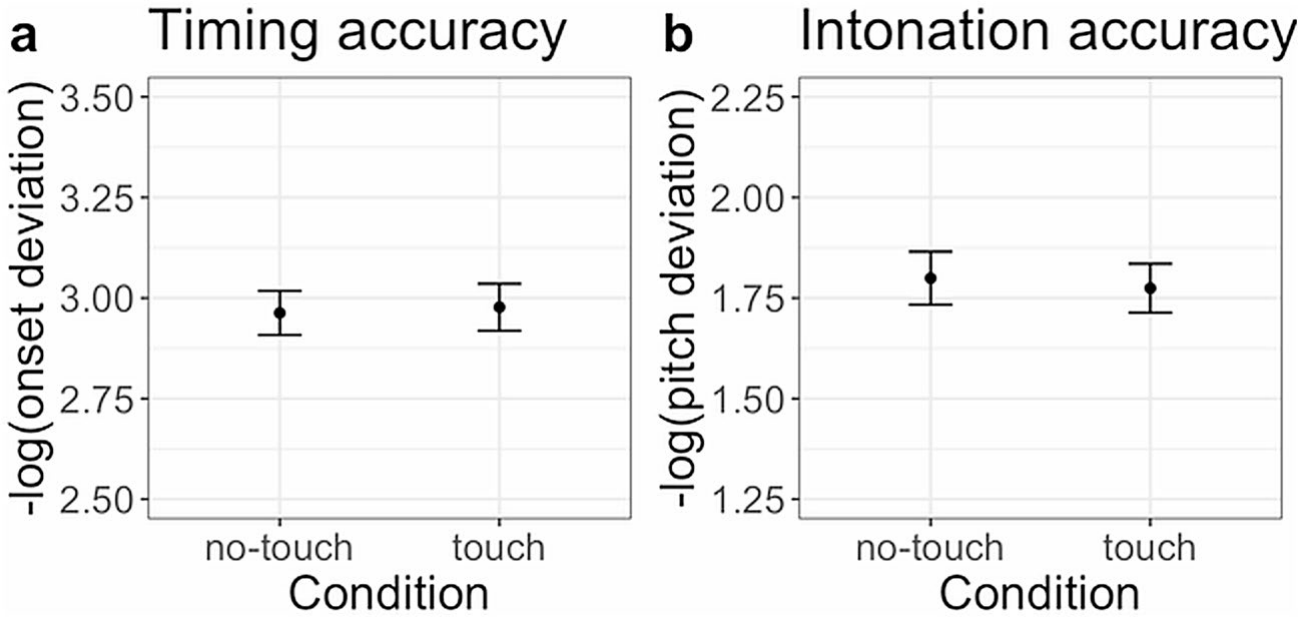
Mean accuracies for timing and intonation across two conditions (touch, no-touch), based on the data analyzed in the *t*-tests. Results did not reveal significant differences. Error bars depict the standard errors of the means.

Comparing mean performances between touch and no-touch by paired *t*-tests did not reveal a significant effect for timing, *t*(31) = 0.90, *p* = .374, *d* = 0.16, 95%-*CI*[−0.20, 0.52], nor for intonation, *t*(31) = ﻿−1.40, *p* = .171, *d* = 0.25, 95%-*CI*[﻿−0.61, 0.11]. Figure 1 depicts the means of these analyses. When fitting the data into linear mixed-effects models, the contrast between touch and no-touch was not significant for both singing quality measures (Table 1). For both models, the best fitting model structure involved voice and piece as random intercepts without slopes [accuracy ~ condition + (1|voice) + (1|piece)]. If anything, there was a tendency for no-touch to decrease intonation accuracy (but *p* > .170 in both approaches). This is opposite to expectation.

**Table 1:** Results of the linear mixed effects models with condition as contrast.

	beta	SE	95% *CI*	*t*	*p*
Timing	0.014	0.018	﻿−0.021, 0.049	0.773	0.441
Intonation	﻿−0.023	0.018	﻿−0.058, 0.011	﻿−1.337	0.183

Neither timing nor intonation revealed significant difference between conditions.

**Fig. 2:**
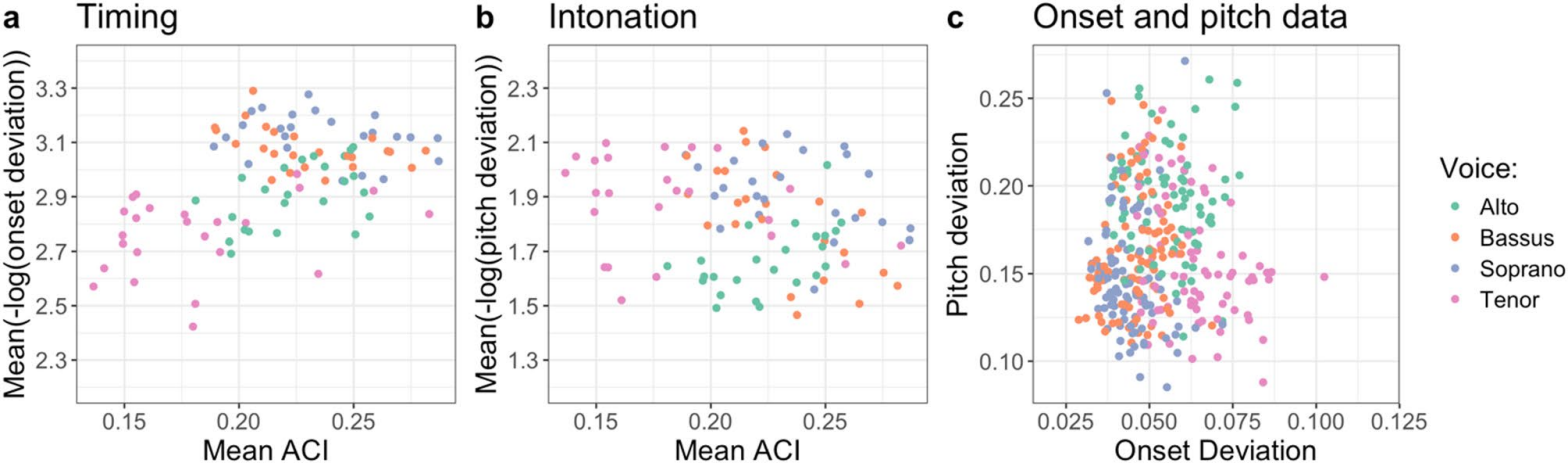
Relation between mean Absolute Coupling Index (ACI) and (a) timing or (**b**) intonation accuracy (96 observations in each plot). Subplot (**c**) shows the relation of between-singer within-voice deviations of onset and pitch in the data set (336 observations, all conditions). Voices are color coded.

Next, we used the respiration coupling index to predict singing quality. Figure 2a indicates an increase in timing accuracy by increased respiration coupling (Mean ACI), but Fig. 2b, if anything, a decrease in pitch accuracy. Splitting the data by voice (see color coding in Fig. 2; see Supplemental Materials, Sect. 3, Figures S1 and S2 depicting results for all three coupling indices) suggests a confound by voice. When taking into account the systematic variance (i.e., voice, block, condition) in five of the six predictive models, the fixed effect of respiration coupling was not significant. The missing fixed effect replicated across all three respiration indices when predicting timing, but only for two of three models when predicting intonation. With PSI as dependent variable, respiration coupling showed a clear but negative effect: Higher coupling resulted in overall lower intonation accuracy. We report all model results in Table 2 (see Supplemental Materials, Sect. 4 and Table S1, for models splitting mean coupling indexes by frequency bands (very low, low or high frequencies) indicating converging results).

**Table 2:** Results of the best fitting models predicting singing accuracy by respiration coupling.

	Accuracy	Best fitting model	beta	*SE*	95% *CI*	*t*	*p*
ACI	Timing	-log(onset deviation) ~ ACI + (1 + ACI|voice) +(1 + ACI|block)	0.430	0.915	﻿−1.737, 2.612	0.470	0.654
	Intonation	-log(pitch deviation) ~ ACI + (1 + ACI|voice) + (1|block) + (1|condition)	﻿−0.861	0.891	﻿−2.989, 1.326	﻿−0.966	0.375
PSI	Timing	-log(onset deviation) ~ PSI + (1 + PSI|voice) +(1|block)	﻿−0.099	0.968	﻿−2.015, 1.818	﻿−0.102	0.923
	Intonation	-log(pitch deviation) ~ PSI + (1|voice) + (1|block) +(1|condition)	﻿−2.179	0.539	﻿−3.256, ﻿−1.107	﻿−4.046	0.0001*
ICI	Timing	-log(onset deviation) ~ ICI + (1 + ICI|voice) +(1 + ICI|block)	﻿−0.126	1.651	﻿−4.089, 3.947	﻿−0.076	0.942
	Intonation	-log(pitch deviation) ~ ICI + (1 + ICI|voice) + (1|block) +(1|condition)	0.276	1.424	﻿−3.235, 3.918	0.194	0.855

Respiration coupling was measured by ACI, PSI, or ICI, singing accuracy in terms of timing or intonation. Note that the PSI intonation model was the only one of the final six models with no random slopes for voice (1 + PSI|voice), but including it did not improve the model fit or change the overall results.

## Discussion

Results across all but one analysis converge: We did not find any evidence for the hypothesis that respiration coupling predicts singing quality. This result is astonishing since breathing directly influences singing performance in pitch, timing, and loudness^46^. We expected higher respiration coupling to improve singing quality. Also, physical touch as a vibrotactile feedback signal between the performers of the ensemble could have improved singing quality^35,36^. Our data does not favor any of these hypotheses. The one significant effect of PSI on intonation accuracy pointed in the other than expected direction.

There are several potential explanations for the null findings.

Even though we based our hypothesis on the assumption that respiration coupling should increase singing quality, studies in other task contexts showed heterogeneous results for the correlation between physiological synchronization and performance synchronization^47^. Correlations were sometimes negative, sometimes positive, and sometimes non-existent. This strongly indicates that the specifics of the studies affected the correlations, such as the chosen physiological measures and tasks. For example, in the study using a joint drumming task^48^, behavioral synchrony correlated with heart beat synchrony. Participants in this study were assembled in groups of three and positioned face-to-face. In contrast, in our study, the singers were positioned in two rows of four, and not everyone could see everyone else. One singer reported afterwards that this had hampered interpersonal communication during the performance. The unusual positioning of the singers might have counteracted a beneficial effect of physiological coupling on singing performance.

The inconclusive results of the meta-analysis^47^ indicated that behavioral synchronization is not tightly linked with physiological synchronization. Research on interpersonal synchronization also deals with its function in social contexts. Here, synchronization in behavior appears to contribute to interpersonal empathy and emotional synchronization (for a review, see^49)^. Some evidence^32^ suggests that also physiological synchronization has socio-affective consequences. Self-rated group cohesion of triads was measured either after joint drumming or joint decision making. Physiological synchronization during the tasks had a positive effect on cohesion and this effect was stable across the tasks. That is, we might expect physiological synchronization during singing to have rather consequences for felt cohesion than improved singing. We did not measure group cohesion in our setting, but in a pilot study, laypersons sang in the same three standing configuration and felt increased bonding by physical contact during singing.

Moreover, synchronization has been demonstrated to change dynamically, instead of being stable across time^48–50^. We calculated the mean coupling index for trials with a duration of 6 min, because it was important to us to capture a valid measure of the low frequencies in the respiration signal for the synchronization index. However, in^16^ we visualized the dynamic changes of physiological coupling between singers also within the 6 min, revealing switches between coupled and decoupled states. This might mirror the musicians’ need to synchronize with others, but also act independently, adjusting the self to the others or promoting specific musical effects, and thereby moving out of synchrony. Such dynamic coordination might be particularly true for singing polyphonic music, as in our study. A finer-grained analysis of the relation between physiological coupling and singing quality could reveal more complex and dynamic dependencies between the two types of synchronization, although this would require careful consideration of the different time scales. For example, Gordon et al.^48^ recorded a physiological signal with a sampling rate of 500 Hz and related this to videos of performance recorded at 30 frames-per-second. To relate both, signals need to be aggregated to the same time window, eventually losing information within each signal. However, the analyses revealed that the positive relation between physiological synchronization and drumming performance fluctuated between stronger and weaker states, even though the overall synchronization within physiology or performance remained rather stable.

Note that a meta-analysis of the relation between physiological synchronization and the relationship quality^47^ revealed distinct outcomes depending on whether the measures related to the parasympathetic or sympathetic nervous system. Based on this observation, we repeated the analyses reported in Table 2, but split the frequency bands into three ranges: very low and low frequency bands for sympathetic contributions, and high frequency bands for parasympathetic contributions (^16, see Supplemental Materials, Section 4, Table S1)^. Importantly, results mostly converged, but model fits seemed to be less reliable: Complex models often failed to converge or were singular. This made the interpretation less reliable. For the PSI and intonation, we found again positive effects for low or high frequencies, but a null effect for very low frequencies. The significant effects for PSI were slightly puzzling. The three respiration measures, ACI, ICI, and PSI, correlated highly, but only ACI and ICI consistently showed null results. However, the PSI takes into account more data with larger phase differences. For ACI and ICI, only relations of close temporal proximity are included. That is, when considering coupling of large phase differences, coupling related to decreased intonation accuracy. It is unclear, why this should be the case. Importantly, the overall conclusion is still valid: Respiration coupling did not increase singing accuracy.

Finally, in general, high performance can result in a ceiling effect, reducing the chance to observe systematic differences. In our study, singing quality was high: mean onset difference between two singers of the same voice was 52 ms (*SD* = 12), mean pitch deviation between two singers of the same voice was 16.4 cents (*SD* = 4). We therefore asked whether our study’s performance level obscured the relationship between physiology and behavior. But, if anything, high performance synchronization seems to enhance such a correlation, as shown by measures of heart beat synchronization during joint drumming^48^. In addition, Fig. 2A−C depicts the variability in the data, which might rule out a simple ceiling effect.

As for most hyperscanning studies, one limitation of the current study is the small sample size. But even though only eight singers were included, the dataset comprised nearly 20 hr of audio data resulting in over 60,000 annotated note events. That is, we regard our measures of intonation and timing as highly reliable. We leave the challenge to scale this up to larger ensembles to future research. The scope of this research is limited to polyphonic Renaissance music, whereas other studies used folk songs, hymns, and canons^17,19,22^, i.e., mainly homophonic compositions, promoting synchronized breathing which in turn boosts the respiration coupling index^22^. For those compositions, it might be even more challenging to relate respiration coupling to singing accuracy, as the synchronous breathing might obscure the delicate relation. A further limitation concerns the absence of vibrato in contemporary performance of Renaissance music^51^. This stylistic decision reduces pitch variability and, in turn, increases pitch accuracy. The use of vibrato is highly individual and, therefore, increases the variability between voices. Only in one short section, the Gloria in Dufay’s “Missa ecce ancilla domini”, we observed a large variation of pitch deviation between the singers towards the end of the musical piece, when the singers became more and more expressive and included vibrato. Investigating music with vibrato might result in more variance of the pitch accuracy, but also poses challenges to the annotation process, as the pitch is naturally less well defined in tones with vibrato.

The observation of physiological coupling between musical performers is a highly intriguing phenomenon, as it eludes conscious control by the members of the network. It was surprising for us that we were unable to uncover a direct beneficial effect of such coupling on the performance quality. Our data do not support the hypothesis that physiological coupling improves singing performance. Future studies might reveal more sophisticated and complex relationships between physiology and performance accuracy in joint music making, including routes via empathy and group cohesion, to provide new insights into processes supporting the production and enjoyment of high-end performance qualities.

## Methods

We report only the necessary information on the method pertaining to our analysis here. For a more detailed report, please see^16^.

### Participants

A professional singing ensemble of Renaissance music (*Cut Circle*) participated in the experiment, with professional singing experience of 4−34 years. The ensemble consisted of eight singers (two women, six men; ages between 29 and 45 years) and one conductor. All participants provided their written informed consent to participate in this study. All procedures were conducted in accordance with the 1964 Helsinki Declaration and its later amendments, and approved by the Ethics Council of the Max Planck Society.

### Apparatus

Singing performances were recorded with individual headset microphones, stereo-AB and surround 5.0 recordings, 48 kHz. We measured physiology with a Brainmap ExG system. Respiration was captured by a respiration belt (BP-BM-10 by BrainProducts).

### Stimulus material

The material consisted of excerpts from Guillaume Dufay’s “Missa ecce ancilla domini” and two motets from Josquin des Prez (see Table 3). To capture low frequencies in the respiration signal, we assembled three recording units of at least 6 min length (“Agnus”, “Kyrie”, “Josquin”). For each unit, we assembled musical pieces including repetitions (e.g., for the recording unit “Josquin”, the ensemble performed the pieces “Virgo prudentissima” – “D’ung aultre amer” – “Virgo prudentissima”). One piece had only two voices (Agnus Dei II), all other pieces were composed for four voices. Each voice was sung by two singers in unison. The polyphonic compositions included imitative passages, and not all voices were always singing all the time. The pieces also included a few homophonic passages with all four voices.

### Procedure

Performance was recorded on three consecutive days. As a warm-up, singers performed all pieces once in the morning of each day. Recordings in different units and spatial arrangements of the ensemble on stage followed. The spatial arrangements were: (i) modern performance practice: each singer sang from his own music stand placed in a semicircle in front of the conductor (“modern”); (ii) resembling historical depictions: singers stood closely in two rows of four in front of one large music stand, with physical contact via putting the hands onto the neighboring singers (“touch”); (iii) same spatial positions of the singers as in (ii) but without physical contact (“no touch”). The singers repeatedly performed the three recording units (i.e., “Agnus”, “Kyrie”, “Josquin”), resulting in a total of eight blocks (three times “Agnus”, two times “Kyrie”, three times “Josquin”, see Table 3). Serial orders of the three recoding units and spatial arrangements were balanced by a complex Latin square. Each of the eight blocks consisted of the three spatial arrangements, resulting in 24 trials in total. We also recorded physiology without singing in two blocks (one on the first, one on the last day of data collection). The number of repeated recordings of musical pieces differed between voices and between pieces (see Table 3). The eight recording units consisted of 25 (alto and tenor) or 31 (bassus and soprano) performed musical sections (including repetitions of pieces) per condition. With each voice sung by two singers and in three spatial arrangements, the audio material added up to 672 files ((31 + 25 + 25 + 31) ⋅ 2 ⋅ 3), and comprising a total of 1,152 min (19.2 h) of music.

**Table 3:** Overview of pieces, recording units, repeated audio recordings for each voice and Spatial arrangement of the ensemble on stage.

Composer	Pieces	Recording Unit [repeats]	Number of repeated audio recordings in each condition (spatial arrangements)			
			Soprano	Altus	Tenor	Bassus
Guillaume Du Fay (Missa ecce ancilla domini)	Agnus I	Agnus [3x]	6	6	6	6
	Agnus II		6	0	0	6
	Agnus III		4	4	4	4
	Kyrie I	Kyrie [2x]	2	2	2	2
	Kyrie II		2	2	2	2
	Gloria		2	2	2	2
Josquin des Prez	Virgo prudentissima	Josquin [3x]	6	6	6	6
	D’ung aultre amer		3	3	3	3
		Blocks: 8	Sum: 31	Sum: 25	Sum: 25	Sum: 31

### Annotation

Annotation is a process to transform the data from an audio file into a symbolic notation. To start with, we created a digital score with *music21*^52^, which represents the “ground truth” or “target”. From the audio files, we extracted onsets, durations, and pitches (measured in Hz) with *Tony*^53^ and enumerated each note event with *Sonic Visualiser*^54^ as the “As-is-state” of the ensemble (for more information see Supplemental Materials, Section 2). The 672 audio recordings contained a total of 65,283 tone events. The automatized extraction was manually reviewed and corrected, if necessary. The extracted raw Hz values of the tone events were converted to fractional MIDI pitch based on concert tuning (a’ = 440 Hz = MIDI pitch 69). Then, a linear regression model over pitch was used to remove pitch drift by substituting raw pitch values with the residual values from the linear regression. For this, we assumed 12-tone equal temperament, which might not be ideal for a cappella performance^55^, as it might employ just intonation. However, it was (post hoc) justified as the measured mean absolute pitch error was 13.6 cents (1/2 tone = 100 cents = 0.5 MIDI pitch), which is smaller than the Pythagorean comma of about 23.46 cents, which denotes the difference between equal and just tuning. We identified gross singing errors by pitch differences of a semitone or more from the score, or onsets differences of more than 300 ms, which was more than the maximum inter-beat interval (250 ms for quarter notes). In total, 1.64% of all sung notes were removed. The final analysis set included 64,214 tone events (for more information on creation of the digital score and the annotation process, please see Supplemental Materials Sects. 1 and 2).

### Data analyses

#### Intonation and timing accuracy

For singing quality, we calculated mean deviations between two singers of the same musical voice. The three recording units (Table 3, third column) consisted of eight music pieces (Table 3, second column), which were repeated two to six times (Table 3, columns 4−7), involving mostly all four voices but one section with two voices only (soprano, bassus). Instead of taking the aggregated recording units as measurement units (which was necessary for the respiration analyses), we took the eight music pieces as measurement units, resulting in 112 data points (= 31 + 25 + 25 + 31) for each spatial arrangement of the ensemble on stage, that is 336 data points in total.

We defined each two measures for intonation and timing, based on the absolute differences of pitches or onsets. Pitch accuracy was measured with Mean Absolute Pitch Error (MAPE), the mean absolute difference in cents between the two singers of each voice across all repetitions of each sung form part. Due to the strongly skewed distribution, we then took the negative logarithm of the MAPE values (LMAPE) to achieve better numerical properties for subsequent analysis. For this negative logarithm, higher values indicate higher accuracy. Similarly, we measured timing precision as the mean absolute onset differences between common tones (MOE) of the two singers in each voice, for each piece. Again, the distribution was strongly skewed and we used the negative logarithm of the MOE values (LMOE), where higher values indicate higher accuracy.

For measuring intonation, we removed event pairs which differed by more than 2.5 semitones, as these are most likely singing or reading errors rather than intonation inaccuracies. This includes two occasions, where the scores were prescribing a temporary split of voices (a minor third in the bassus and a fifth in the soprano of Dufay’s Agnus I). This reduced the original number of 31,421 by 57 (0.2%) to 31,364 final event pairs.

For timing accuracy analyses, we removed cases where the tone onsets differed by more than 300 ms, as these were deemed singing errors rather than imprecise timing. The criterion is roughly the double of the usual 1.5 ⋅ *IQR* outlier criterion, which was 148 ms, which seemed too short for musical reasons. This removed 114 from 31,421 event pairs (0.4%) to a final total of 31,307.

Note that, whereas the respiration data in^16^ had to be clipped at 360 s duration for methodological reasons, we included audio recordings in full length in these analyses to maximize measurement reliability of the mean deviations. For example, onset deviations between singers could increase, decrease, a mix of both, or stay the same, depending on the affordances of the musical piece. Then, the more data we use to average across, the more reliable the mean measurement of performance errors will be.

#### Respiration coupling

We computed phases in the frequency range 0.025–0.40 Hz by a Morlet wavelet transform and phase differences were computed from the wavelet coefficients for all possible subjects/channel pairs. We chose ten frequency components and defined phase differences in the range of -π/4 and + π/4 as being coupled. The ACI counts the relative number of phase-locked points within this range (see^16^ for more details). In addition, we repeated analyses for two other indices, the PSI and the ICI, an asymmetric coupling measure built from the ACI and phase-locked points within the positive range only. However, the three measures are highly correlated: ACI and PSI with *r* = .81, ACI and ICI with *r* = .92, PSI and ICI with *r* = .66, all *p* < .0001. As a consequence, we assumed the models not to differ strongly between the three measures. The recording units were clipped at 360 s for this analysis. The data consisted of ACIs analyzed from the eight recording units of eight singers for ten frequencies (640 data points) for each spatial arrangement. The coupling index included coupling of each singer to all other singers. The underlying rationale is that during singing, each singer will also relate to the other singers and not only to the one in the same voice, so all relations were included. For the respiration coupling index of each voice, we averaged the individual coupling indices of the two singers of each voice.

#### Statistical analyses

Analysis was based on *N* = 4 voices for the within-voice accuracy measures and *N* = 8 singers for the respiration data, calculating synchronization between each singer and the other singers. We took care to adjust analyses between measures as best as possible and present here more than one way to analyze the data to aim for a converging and reliable interpretation. The data set on accuracies included a total of 336 observations (112 in each spatial arrangement of the ensemble). Importantly, each observation was an average across the between-singer within-voice deviation of accuracy for one musical unit. That is, data were not raw data but means, increasing the reliability of each observation, which was particularly important because of the low number of cases.

#### Comparisons of mean singing accuracy with touch and without touch

Based on the effect of touch on respiration coupling^16^ and the strong dependencies between motor control of respiration and singing, we expected singing accuracy to increase by touch. We compared singing accuracy between conditions by two approaches and included data from the two spatial arrangements touch and no-touch (112 ⋅ 2 = 224 observations).

The first approach was via paired *t*-tests, to resemble the analyses reported in^16^. We applied the *t.test()* function from the *stats* package^56^ and the *cohen.d()* function from the *effsize* package^57^. Instead of eight singers, our comparison was based on voices. Averaging across all musical units would result in *N* = 4 paired observations (four voices). We therefore divided the data on the level of blocks to increase the number of observations for analyses. The 24 trials of audio and physiological recordings were arranged in eight blocks. Each block included the three spatial arrangements of the ensemble for one of the three recording units (“Agnus”, “Kyrie”, “Josquin”). That is, we recorded all three arrangements of one recording unit (e.g., “Agnus”) before we continued with a different unit (e.g., “Kyrie”). We created means (for voices and arrangements) separately for the eight blocks. We treated the spatial arrangements in each block as within-subject (voice) variable, but the eight blocks as between-subject variable (even though they were within-subject repeated measures). That is, collapsing across the 224 observations resulted in *n* = 32 paired observations of mean accuracies (eight blocks x four voices). Treating the within-subject measure of block as between-subject variable decreased the power to detect a true effect. We therefore also report a second approach applying linear mixed effects modelling with contrast coding of the two spatial arrangements and implementing voice and blocks as random intercepts, as well as pieces, which were the smallest units in the audio data (mean accuracy for “Agnus Dei I”, Agnus Dei II” etc.). Maximal models included random slopes and did not converge. Iterative model comparison started with models including all random intercepts. Data for the models included all 224 observations and were fitted using the *lme4* package in R^58^, assuming significance for *t* > 2, but *p*-estimates are also given using the *lmerTest* package^59^.

#### Predicting singing quality by respiration coupling

We expected synchronized respiration to causally increase singing accuracy because of the dependencies of the two signals. For the analyses, we had to adjust the units of the singing quality measures. Whereas we had based the analysis of singing quality on musical pieces, the respiration coupling data required longer recording units to capture very low frequencies in the signal. We therefore decided on the recording units (see Table 3) as units for both measures, resulting in 96 observations (four voices, eight recording units, three choir setups). Note that we added data from the third spatial arrangement here (see Procedure, (i) modern performance practice), which had been neglected in our analyses so far, as it functioned as a control measure to compare this normal singing situation with a resting condition without singing, reported in the original publication^16^. We fit the data with linear mixed-effects models. Respiration coupling was predicted by either pitch (-log(absolute pitch difference)) or onset accuracy (-log(absolute onset difference)), with voice, block, and condition as random intercepts. We opted to start with the full model, which did not fit for both dependent variables. We thereby reduced the number of parameters and started with LMOE ~ ACI + (1|voiceF) + (1 + ACI|blockF) + (1 + ACI|conF) for the onset model and with LMAPE ~ ACI + (1 + ACI|voiceF) + (1 + ACI|blockF) + (1|conF) for the pitch model. We further reduced the number of parameters based on model comparisons using likelihood-ratio tests^60^ and report the best fitting model with the lowest number of parameters.

## Supplementary Information

Below is the link to the electronic supplementary material.

Supplementary Material 1

## Data Availability

The annotated data were processed by scripts available at https://github.com/klausfrieler/cut_circle_voice_data. The resulting preprocessed mean data (336 observations) and all scripts with statistical analyses are available at https://osf.io/w7vhu/.

## References

[1] D’Ausilio, A., Novembre, G., Fadiga, L. & Keller, P. E. What can music tell us about social interaction? *Trends Cognit Sci.* **19**, 111–114. 10.1016/j.tics.2015.01.005 (2015). 25641075 10.1016/j.tics.2015.01.005

[2] MacCormick, L. Routledge,. Music as social performance in *Myth, Meaning, and Performance*, (eds R. Eyerman & L. MacCormick) pp. 121–144. (2016).

[3] Volpe, G., D’Ausilio, A., Badino, L., Camurri, A. & Fadiga, L. Measuring social interaction in music ensembles. *Philos. Trans. R. Soc. Lond. B Biol. Sci.* 371. 10.1098/rstb.2015.0377 (2016). 10.1098/rstb.2015.0377 PMC4843615 27069054

[4] Palmer, C., Spidle, F., Koopmans, E. & Schubert, P. Ears, heads, and eyes: when singers synchronise. *Q. J. Experimental Psychol.* **72**, 2272–2287. 10.1177/1747021819833968 (2019). 10.1177/1747021819833968 30744490

[5] Acquadro, M. A. S., Congedo, M. & de Riddeer, D. Music performance as an experimental approach to hyperscanning studies. *Front. Hum. Neurosci.* **10**, 242. 10.3389/fnhum.2016.00242 (2016). 27252641 10.3389/fnhum.2016.00242 PMC4879135

[6] Cheng, S., Wang, J., Luo, R. & Hao, N. Brain to brain musical interaction: a systematic review of neural synchrony in musical activities. *Neurosci. Biobehav Rev.* **164**, 105812. 10.1016/j.neubiorev.2024.105812 (2024). 39029879 10.1016/j.neubiorev.2024.105812

[7] Lindenberger, U., Li, S. C., Gruber, W. & Müller, V. Brains swinging in concert: cortical phase synchronization while playing guitar. *BMC Neurosci.* **10**, 22. 10.1186/1471-2202-10-22 (2009). 19292892 10.1186/1471-2202-10-22 PMC2662862

[8] Sänger, J., Müller, V. & Lindenberger, U. Directionality in hyperbrain networks discriminates between leaders and followers in guitar duets. *Front. Hum. Neurosci.* **7**, 234. 10.3389/fnhum.2013.00234 (2013). 23761745 10.3389/fnhum.2013.00234 PMC3671173

[9] Sänger, J., Müller, V. & Lindenberger, U. Intra- and interbrain synchronization and network properties when playing guitar in duets. *Front. Hum. Neurosci.* **6**, 312. 10.3389/fnhum.2012.00312 (2012). 23226120 10.3389/fnhum.2012.00312 PMC3509332

[10] Gugnowska, K. et al. Endogenous sources of interbrain synchrony in duetting pianists. *Cereb. Cortex*. **32**, 4110–4127. 10.1093/cercor/bhab469 (2022). 35029645 10.1093/cercor/bhab469 PMC9476614

[11] Lender, A., Perdikis, D., Gruber, W., Lindenberger, U. & Müller, V. Dynamics in interbrain synchronization while playing a piano duet. *Ann. N Y Acad. Sci.* **1530**, 124–137. 10.1111/nyas.15072 (2023). 37824090 10.1111/nyas.15072

[12] Zamm, A. et al. Amplitude envelope correlations measure synchronous cortical oscillations in performing musicians. *Ann. N Y Acad. Sci.*10.1111/nyas.13738 (2018). 29756657 10.1111/nyas.13738

[13] Zamm, A. et al. Behavioral and neural dynamics of interpersonal synchrony between performing musicians: a wireless EEG hyperscanning study. *Front. Hum. Neurosci.* **15**, 717810. 10.3389/fnhum.2021.717810 (2021). 34588966 10.3389/fnhum.2021.717810 PMC8473838

[14] Delius, J. A. M. & Müller, V. Interpersonal synchrony when singing in a choir. *Front. Physiol.* **13**, 1087517. 10.3389/fpsyg.2022.1087517 (2023). 10.3389/fpsyg.2022.1087517 PMC9875726 36710769

[15] Hemakom, A., Goverdovsky, V., Aufegger, L. & Mandic, D. P. Quantifying cooperation in choir singing: respiratory and cardiac synchronisation *IEEE International Conference on Acoustics, Speech and* Signal Processing *(ICASSP)*, pp. 719–723, 10.1109/ICASSP.2016.7471769 (2016).

[16] Lange, E. B. et al. In touch: cardiac and respiratory patterns synchronize during ensemble singing with physical contact. *Front. Hum. Neurosci.* **16**, 928563. 10.3389/fnhum.2022.928563 (2022). 35992947 10.3389/fnhum.2022.928563 PMC9390082

[17] Müller, V., Delius, J. A. M. & Lindenberger, U. Complex networks emerging during choir singing. *Ann. N Y Acad. Sci.* **1431**, 85–101. 10.1111/nyas.13940 (2018). 30058160 10.1111/nyas.13940

[18] Müller, V., Delius, J. A. M. & Lindenberger, U. Hyper-frequency network topology changes during choral singing. *Front. Physiol.* **10**, 207. 10.3389/fphys.2019.00207 (2019). 30899229 10.3389/fphys.2019.00207 PMC6416178

[19] Müller, V. & Lindenberger, U. Cardiac and respiratory patterns synchronize between persons during choir singing. *PloS One*. **6**, e24893. 10.1371/journal.pone.0024893 (2011). 21957466 10.1371/journal.pone.0024893 PMC3177845

[20] Osaka, N. et al. How two brains make one synchronized Mind in the inferior frontal cortex: fNIRS-based hyperscanning during cooperative singing. *Front. Psychol.* **6**, 1811. 10.3389/fpsyg.2015.01811 (2015). 26635703 10.3389/fpsyg.2015.01811 PMC4659897

[21] Ruiz-Blais, S., Orini, M. & Chew, E. Heart rate variability synchronizes when non-experts vocalize together. *Front. Physiol.* **11**, 762. 10.3389/fphys.2020.00762 (2020). 33013429 10.3389/fphys.2020.00762 PMC7506073

[22] Vickhoff, B. et al. Music structure determines heart rate variability of singers. *Front. Psychol.* **4**, 334. 10.3389/fpsyg.2013.00334 (2013). 23847555 10.3389/fpsyg.2013.00334 PMC3705176

[23] Babiloni, C. et al. Brains in concert: frontal oscillatory alpha rhythms and empathy in professional musicians. *NeuroImage* **60**, 105–116. 10.1016/j.neuroimage.2011.12.008 (2012). 22186679 10.1016/j.neuroimage.2011.12.008

[24] Greco, A. et al. EEG hyperconnectivity study on saxophone quartet playing in ensemble. *40th Annual International Conference of the IEEE Engineering in Medicine and Biology Society*. 10.1109/embc.2018.8512409 (2018). 10.1109/EMBC.2018.8512409 30440563

[25] Ramírez-Moreno, M. A. et al. Brain-to-brain communication during musical improvisation: a performance case study. *F1000Research* **11**, 989. 10.12688/f1000research.123515.4 (2022). 37809054 10.12688/f1000research.123515.4 PMC10558998

[26] Müller, V. & Lindenberger, U. Dynamic orchestration of brains and instruments during free guitar improvisation. *Front. Integr. Neurosci.* **13**10.3389/fnint.2019.00050 (2019). 10.3389/fnint.2019.00050 PMC6738335 31551723

[27] Müller, V. & Lindenberger, U. Probing associations between interbrain synchronization and interpersonal action coordination during guitar playing. *Ann. N Y Acad. Sci.* **1507**, 146–161. 10.1111/nyas.14689 (2022). 34510474 10.1111/nyas.14689

[28] Müller, V., Fairhurst, M. T., van Vugt, F. T., Keller, P. E. & Müller, M. F. Editorial: interpersonal synchrony and network dynamics in social interaction. *Front. Hum. Neurosci.* **16**, 1095735. 10.3389/fnhum.2022.1095735 (2022). 36523443 10.3389/fnhum.2022.1095735 PMC9745327

[29] Abalde, S. F., Rigby, A., Keller, P. E. & Novembre, G. A framework for joint music making: behavioral findings, neural processes, and computational models. *Neurosci. Biobehav Rev.* **167**, 105816. 10.1016/j.neubiorev.2024.105816 (2024). 39032841 10.1016/j.neubiorev.2024.105816

[30] Novembre, G., Sammler, D. & Keller, P. E. Neural alpha oscillations index the balance between self-other integration and segregation in real-time joint action. *Neuropsychologia* **89**, 414–425. 10.1016/j.neuropsychologia.2016.07.027 (2016). 27449708 10.1016/j.neuropsychologia.2016.07.027

[31] Gordon, I. et al. Physiological and behavioral synchrony predict group cohesion and performance. *Sci. Rep.* **10**, 8484. 10.1038/s41598-020-65670-1 (2020). 32439861 10.1038/s41598-020-65670-1 PMC7242382

[32] Tomashin, A., Gordon, I. & Wallot, S. Interpersonal physiological synchrony predicts group cohesion. *Front. Hum. Neurosci.* **16**, 903407. 10.3389/fnhum.2022.903407 (2022). 35903785 10.3389/fnhum.2022.903407 PMC9314573

[33] Watson, A. Breathing in singing In *The oxford handbook of singing*. (eds G. F. Welch, D. M. Howard, J. Nix & A. Watson 10.1093/oxfordhb/9780199660773.013.10 pp. 86–108. (Oxford University Press, 2019).

[34] Salomoni, S., van den Hoorn, W. & Hodges, P. Breathing and singing: objective characterization of breathing patterns in classical singers. *PloS One*. **11**, e0155084. 10.1371/journal.pone.0155084 (2016). 27159498 10.1371/journal.pone.0155084 PMC4861272

[35] Sundberg, J. Phonatory vibrations in singers: a critical review. *Music Percept.* **9**, 361–381. 10.2307/40285557 (1992).

[36] Russo, F. A., Ammirante, P. & Fels, D. I. Vibrotactile discrimination of musical timbre. *J. Exp. Psychol. Hum. Percept. Perform.* **38**, 822–826. 10.1037/a0029046 (2012). 22708743 10.1037/a0029046

[37] Harrison, S. J. & Richardson, M. J. Horsing around: spontaneous four-legged coordination. *J. Mot Behav.* **41**, 519–524. 10.3200/35-08-014 (2009). 19567365 10.3200/35-08-014

[38] Lagarde, J. & Kelso, J. A. S. Binding of movement, sound and touch: multimodal coordination dynamics. *Exp. Brain Res.* **173**, 673–688. 10.1007/s00221-006-0410-1 (2006). 16528497 10.1007/s00221-006-0410-1

[39] Sofianidis, G., Hatzitaki, V., Grouios, G., Johannsen, L. & Wing, A. Somatosensory driven interpersonal synchrony during rhythmic sway. *Hum. Mov. Sci.* **31**, 553–566. 10.1016/j.humov.2011.07.007 (2012). 22742723 10.1016/j.humov.2011.07.007

[40] Repp, B. H. Probing the cognitive representation of musical time: structural constraints on the perception of timing perturbations. *Cognition* **44**, 241–281. 10.1016/0010-0277(92)90003-z (1992). 1424494 10.1016/0010-0277(92)90003-z

[41] Repp, B. H. A constraint on the expressive timing of a melodic gesture: evidence from performance and aesthetic judgment. *Music Percept.* **10**, 221–241. 10.2307/40285608 (1992).

[42] Larrouy-Maestri, P., Lévêque, Y., Schön, D., Giovanni, A. & Morsomme, D. The evaluation of singing voice accuracy: a comparison between subjective and objective methods. *J. Voice*. **27**, 259e1. 10.1016/j.jvoice.2012.11.003 (2013). 259.e5. 10.1016/j.jvoice.2012.11.003 23280380

[43] Alldahl, P. G. *Choral Intonation*. (Gehrmans Musikförlag, 2008).

[44] Mauch, M., Frieler, K. & Dixon, S. Intonation in unaccompanied singing: accuracy, drift, and a model of reference pitch memory. *J. Acoust. Soc. Am.* **136**, 401–411. 10.1121/1.4881915 (2014). 24993224 10.1121/1.4881915

[45] Seaton, R., Pim, D. & Sharp, D. Pitch drift in a cappella choral singing. *Proc. Inst. Acoust.* **35**, 358–364 (2013). https://oro.open.ac.uk/38140

[46] Welch, G. F., Howard, D. M., Nix, J. & Watson, A. (eds) *The Oxford Handbook of Singing* (Oxford University Press, 2019).

[47] Mayo, O., Lavidor, M. & Gordon, I. Interpersonal autonomic nervous system synchrony and its association to relationship and performance - a systematic review and meta-analysis. *Physiol. Behav.* **235**, 113391. 10.1016/j.physbeh.2021.113391 (2021). 33744259 10.1016/j.physbeh.2021.113391

[48] Gordon, I., Gilboa, A., Cohen, S. & Kleinfeld, T. The relationship between physiological synchrony and motion energy synchrony during a joint group drumming task. *Physiol. Behav.* **224**, 113074. 10.1016/j.physbeh.2020.113074 (2020). 32663553 10.1016/j.physbeh.2020.113074

[49] Mayo, O. & Gordon, I. In and out of synchrony-behavioral and physiological dynamics of dyadic interpersonal coordination. *Psychophysiology* **57**, e13574. 10.1111/psyp.13574 (2020). 32221984 10.1111/psyp.13574

[50] Gordon, I., Tomashin, A. & Mayo, O. A theory of flexible multimodal synchrony. *Psychol. Rev.* **132**, 680–718. 10.1037/rev0000495 (2025). 39446615 10.1037/rev0000495

[51] Phillips, P. Singing polyphony. *The Musical Times*, 7–18 https://www.jstor.org/stable/24615654 (2014).

[52] Cuthbert, M., Ariza, C. & Hogue, B. & Oberholtzer, Josiah, Wolf. *Music21* (Software). https://www.music21.org/

[53] Mauch, M. et al. S *Computer-aided melody note transcription using the tony software: Accuracy and efficiency*. https://code.soundsoftware.ac.uk/attachments/download/1423/tony-paper_preprint.pdf (2015).

[54] Cannam, C., Landone, C. & Sandler, M. Sonic Visualiser: an open source application for viewing, analysing, and annotating music audio files. In *Proceedings of the ACM Multimedia 2010 International Conference* 1467–1468 http://portal.acm.org/citation.cfm?id=1873951.1874248 (2010).

[55] Howard, D. M. Intonation drift in a capella soprano, alto, tenor, bass quartet singing with key modulation. *J. Voice*. **21**, 300–315. 10.1016/j.jvoice.2005.12.005 (2007). 16527450 10.1016/j.jvoice.2005.12.005

[56] R Core Team. R: a language and environment for statistical computing. https://www.r-project.org/ (2025).

[57] Torchiano, M. *Effsize - A package for efficient effect size computation*. 10.5281/zenodo.196082 (2016).

[58] Bates, D., Mächler, M., Bolker, B. & Walker, S. Fitting linear mixed-effects models using lme4. *J. Stat. Soft*. **67**10.18637/jss.v067.i01 (2015).

[59] Kuznetsova, A., Brockhoff, P. B. & Christensen, R. H. B. LmerTest package: tests in linear mixed effects models. *J. Stat. Soft*. **82**10.18637/jss.v082.i13 (2017).

[60] Baayen, R. H., Davidson, D. J. & Bates, D. M. Mixed-effects modeling with crossed random effects for subjects and items. *J. Mem. Lang.* **59**, 390–412. 10.1016/j.jml.2007.12.005 (2008).

