# Supplementary material for: Intonation and timing in singing early music is unrelated to respiration synchronization
Source: Sci Rep. 2026 Feb 27;16:7834. doi: 10.1038/s41598-026-39565-6 (PMC12953873; doi:10.1038/s41598-026-39565-6)
Supplement: Supplementary file 1 — Supplementary Material 1 [file 41598_2026_39565_MOESM1_ESM.docx]

**Supplemental Materials**

To the manuscript “Synchronization of the respiration signal between singers is unrelated to singing quality” by

Anton Schreiber, Klaus Frieler, Elke Lange

to be published in *Scientific Reports*

(February 3^rd^, 2026)

Content

- Section 1: Creation of the digital score [*music21*]
- Section 2: Annotation of the audio files [*Tony*, *Sonic Visualiser*]
- Section 3: Predicting singing accuracy from coupling of the respiration signal [including Figures S1 and S2, depicting the results of the linear mixed effects models for the respiration coupling indices ACI, PSI, and ICI]
- Section 4: Splitting frequency bands into different ranges to separately look into effects of parasympathetic or sympathetic coupling [including Table S1]

## **Section 1: Creation of the digital score**

We used the *Python* package *music21*^[1]^ to annotate the sheet music as .xml files of all sung pieces. We wrote a function that enumerated every note event for each voice of the .xml score for each piece, starting with one for the first note until the last note of the section. In case of several options for one note event, each note was annotated with an alphabetical letter (e.g., 25a). To extract the pitch and timing information of the score, we wrote a second function with the package music21 that extracted 1) note label 2) voice 3) onset in quarter notes 4) measure number 5) position of the note in bar 6) MIDI-pitch 7) note duration in quarter notes, and 8) time signature. The resulting data represented the “To-be-state” with which we compared the “As-is-state” of the ensemble.

## **Section 2: Annotation of the audio files**

The .wav recording of every take for every singer was note-tracked with the software *Tony*^[2^^]^ and exported as CSV files with onset, duration, and precise f0 values converted to cents. The annotation of the software were manually checked and adjusted, if needed. This included : 1) adding undetected notes, 2) splitting wrongly fused notes, and 3) adjusting incorrect note pitches, onsets or durations. The CSV were imported into *Sonic Vizualizer*^[3]^ as annotation layers to carry out the subsequent manual annotations in a convenient manner. To this end, every note event was annotated with a number corresponding to the number of the note event from the original score to be able to identify the intention of the singers. Furthermore, to assess performance errors, we also annotated 1) incorrectly sung notes, 2) additional tones that were not part of the score, 3) interrupted notes due to breathing, 4) improvised notes, that were sung in substitution for the notes written in the score.

## **Section 3: Predicting singing accuracy from coupling of the respiration signal**

We analyzed in the main text the relation between respiration coupling and singing accuracy by a modeling approach, using linear mixed-effects models. A total of six models were fitted, including three different respiration coupling measures (ACI, PSI, ICI) and two measures of singing quality (timing and intonation). The model predicting intonation accuracy by PSI showed in a significant effect. Interestingly, the effect was in the opposite direction than predicted. Higher coupling as measured by PSI resulted in lower intonation accuracy. Figure S1 depicts the original data for all six analyses.

In addition, we compared the original data with the model-adjusted means by the *remef* function^[4]^ in R in Figure S2). The function adjusts the means for the fitted random effects in the data and thereby shows the data on which the fixed effect of respiration coupling is based on. Whereas including random effects in the models on ACI and ICI result in flat regression lines for all voices, it results in negative slopes for the PSI in all four voices. We cannot offer any explanation, why the significant effect occurred for this specific model.


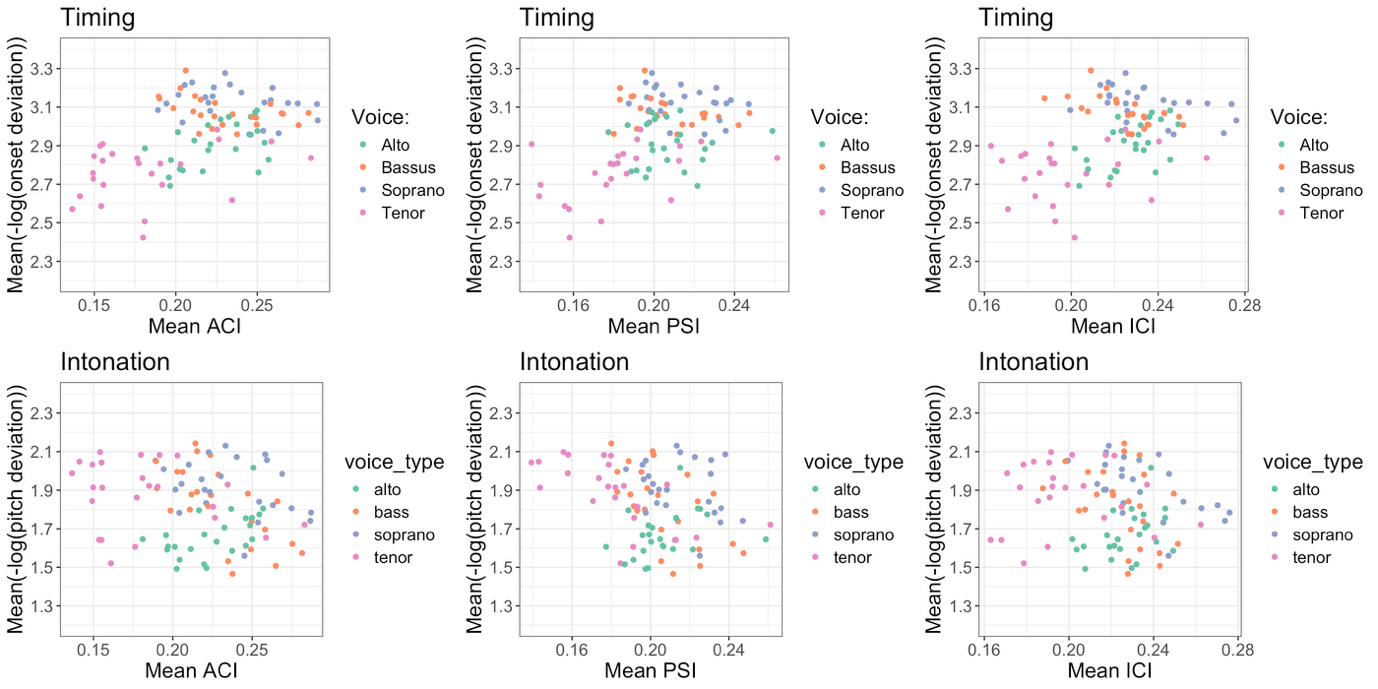


**Figure S1.** Comparisons of relations between respiration coupling and timing or intonation for three coupling measures


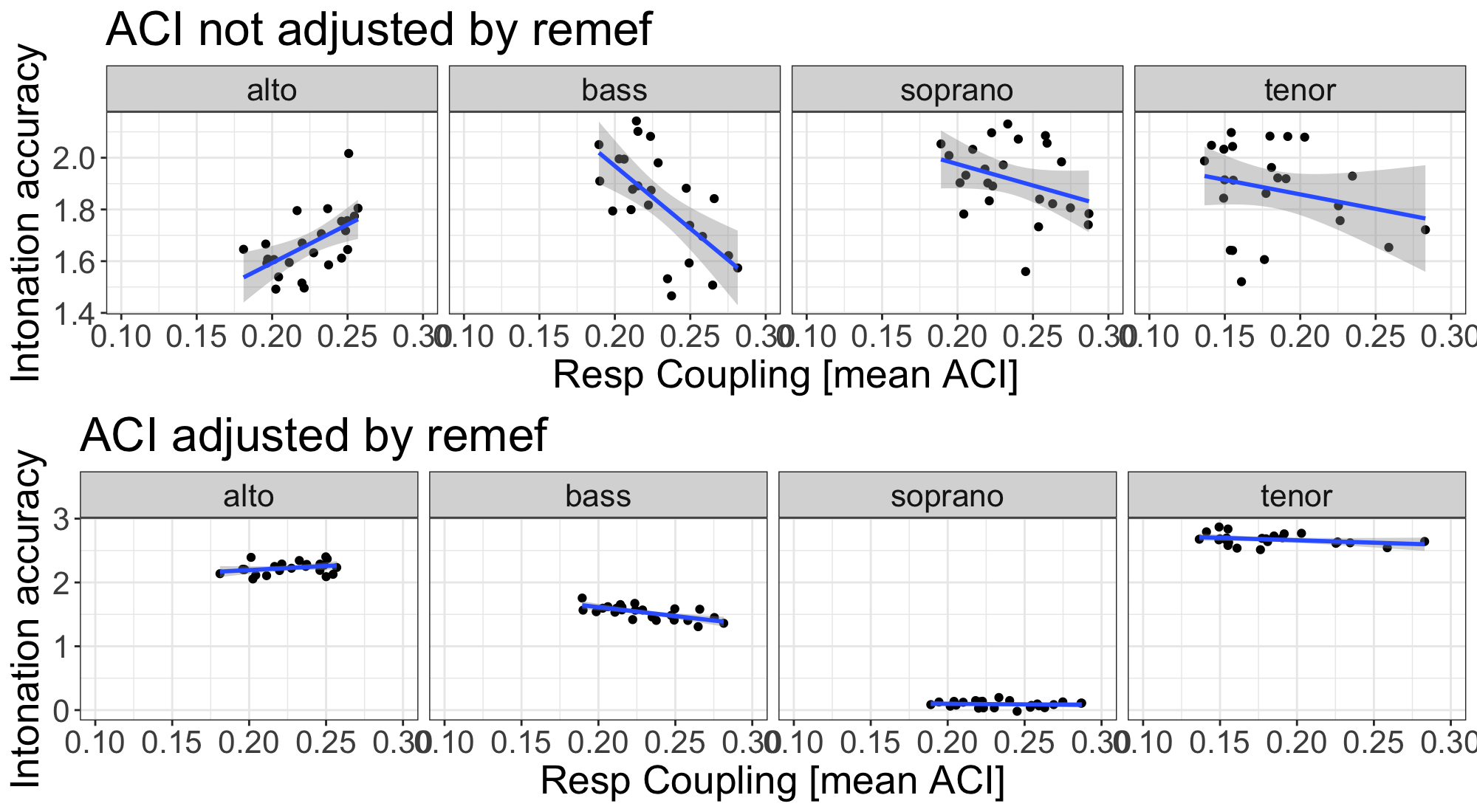


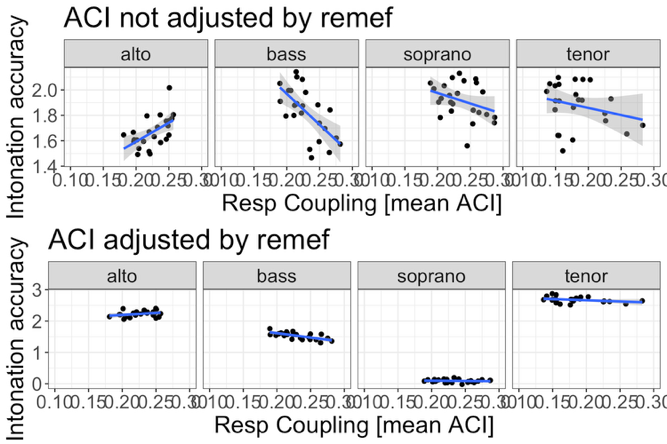

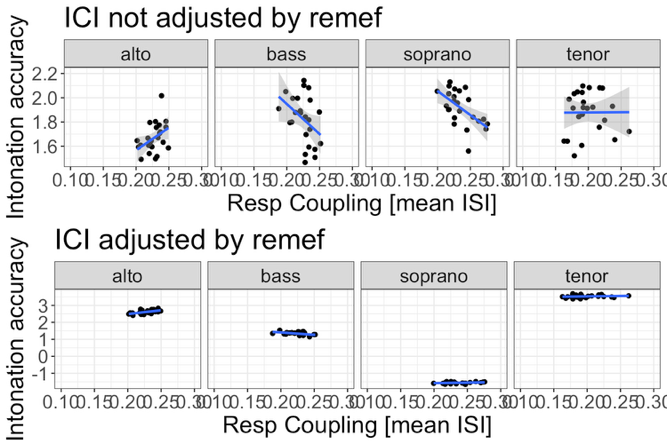


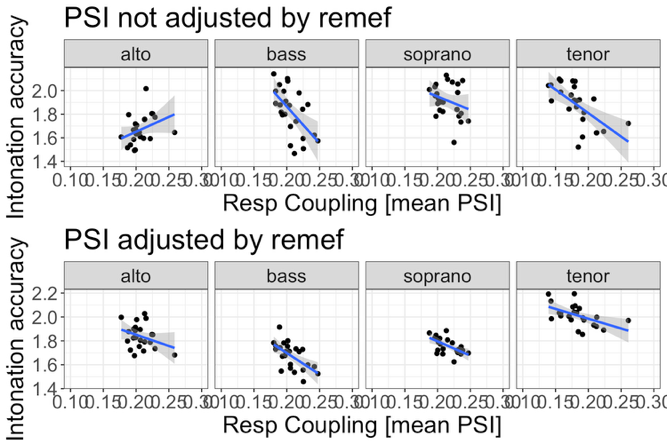


**Figure S2.** Intonation accuracy predicted by raw or model-adjusted means of three respiration coupling indices.

**Section 4: Splitting frequency bands into different ranges to separately look into effects of parasympathetic or sympathetic coupling**

It has been shown that the correlations between physiological synchrony and the quality of relationship differed depending on the physiological ranges that support the parasympathetic or the sympathetic nervous system.^[5]^ No such effect has been reported for the correlation between physiological synchrony and performance synchrony. Nevertheless, we repeated model fitting (Table 2, main text) for three frequency ranges of coupling. Very low frequencies (VLF: 0.025, 0.05 Hz) and low frequencies (LF: 0.075, 0.10, 0.125, 0.15 Hz) support the sympathetic nervous system, and high frequencies (HF: 0.20, 0.25, 0.30, 0.40 Hz) the parasympathetic.

Results mostly converged with the model fits, when frequency bands were averaged (Table 2, main text). But for the ACI-VLF model on timing, the best fitting model with the least number of parameters showed a positive effect. However, it was a very simple model. In contrast, the next best fitting model included 5 more parameters and resulted in a non-significant main effect (model: ACI_VLF + (1+ACI_VLF|voiceF) + (1+ACI_VLF|blockF), *t* = 1.44, *p* = .202). During the iterative model fitting procedure, models failed to fit or produced singular fits very often, indicating that models were overpowered by including too many random intercepts and slopes (particularly for the timing models). Therefore, we would regard this analysis as not highly reliable, and results in Table S1 have to be interpreted with caution. The general tendency, however, is that results do not really differ for different frequency ranges.

**Table S1.**

***Model results taking into account ranges of frequency bands***

|  |  |  | Best fitting model | *t* | *p* |
| --- | --- | --- | --- | --- | --- |
| ACI | Timing | VLF | **~ ACI_VLF + (1\|voiceF)** | **2.919** | **0.004** |
|  |  | LF | ~ ACI_LF + (1\|voiceF) + (1\|blockF) | 0.808 | 0.425 |
|  |  | HF | ~ ACI_HF + (1+ACI_HF\|voiceF) + (1+ACI_HF\|blockF) | 0.328 | 0.753 |
|  | Intonation | VLF | ~ ACI_VLF + (1+ACI_VLF\|voiceF) + (1\|blockF) + (1\|conF) | 0.002 | 0.999 |
|  |  | LF | ~ ACI_LF + (1+ACI_LF\|voiceF) + (1\|blockF) + (1\|conF) | -1.16 | 0.299 |
|  |  | HF | ~ ACI_HF + (1+ACI_HF\|voiceF) + (1\|blockF) + (1\|conF) | -0.867 | 0.422 |
| PSI | Timing | VLF | ~ PSI_VLF + (1\|voiceF) | 1.657 | 0.101 |
|  |  | LF | ~ PSI_LF + (1\|voiceF) | 1.275 | 0.205 |
|  |  | HF | ~ PSI_HF + (1\|voiceF) | -0.762 | 0.448 |
|  | Intonation | VLF | ~ PSI_VLF + (1\|voiceF) + (1\|blockF) + (1\|conF) | -1.077 | 0.284 |
|  |  | **LF** | **~ PSI_LF + (1\|voiceF) + (1\|blockF) + (1\|conF)** | **-3.718** | **0.004** |
|  |  | **HF** | **~ PSI_HF + (1\|voiceF) + (1\|blockF) + (1\|conF)** | **-2.736** | **0.008** |
| ICI | Timing | VLF | ~ ICI_VLF + (1\|voiceF) | 1.513 | 0.134 |
|  |  | LF | ~ ICI_LF + (1+ICI_LF\|voiceF) + (1+ICI_LF\|blockF) | -0.530 | 0.615 |
|  |  | HF | ~ ICI_HF + (1+ICI_HF\|voiceF) + (1+ICI_HF\|blockF) | 0.091 | 0.931 |
|  | Intonation | VLF | ~ ICI_VLF + (1\|voiceF) + (1\|blockF) + (1\|conF) | -0.912 | 0.364 |
|  |  | LF | ~ ICI_LF + (1\|voiceF) + (1\|blockF) + (1\|conF) | -0.097 | 0.923 |
|  |  | HF | ~ ICI_HF + (1+ICI_HF\|voiceF) + (1\|blockF) + (1\|conF) | 0.511 | 0.635 |

Note: The dependent variable for timing was -log(onset deviation) and for intonation -log(pitch deviation). Significant models are highlighted in bold font.

**References**

1. Cuthbert, M., Ariza, C., Hogue, B. & Oberholtzer, Josiah, Wolf. *Music21* (Software).

2. Mauch, M. et al. Computer-aided melody note transcription using the tony software: Accuracy and efficiency. *Proceedings of the first International Conference on Technologies for Music Notation and Representation (TENOR)*, Paris, France (2015).

3. Cannam, C., Landone, C. & Sandler, M. Sonic Visualiser: An open source application for viewing, analysing, and annotating music audio files. *Proceedings of the ACM Multimedia 2010 International Conference,* Firenze, Italy, 1467–1468 (2010).

4. Hohenstein, S. & Kliegel, R. *Remef (REMove effects)* (Software, version v0.6.10). Retrieved from https://read.psych.uni-potsdam.de/joomla/attachments/article/12/remef.v0.6.10.R (2013).

5. Mayo, O., Lavidor, M. & Gordon, I. Interpersonal autonomic nervous system synchrony and its association to relationship and performance - a systematic review and meta-analysis. *Physiology & Behavior* **235,** 113391 (2021).
